# Barriers to Inclusive Sexual and Reproductive Health Care for LGBTQ+ Populations: A Narrative Review With Emphasis on Midwifery and Obstetric Services in Greece

**DOI:** 10.7759/cureus.110161

**Published:** 2026-06-03

**Authors:** Zoi Koukou, Efstathia Davidia Stergiou

**Affiliations:** 1 Department of Midwifery, International Hellenic University, Thessaloniki, GRC

**Keywords:** access to services, discrimination, education, health care staff, heteronormativity, lgbtq+, midwives, obstetric, reproductive health, sexual health

## Abstract

Despite recent legal and policy advancements, LGBTQ+ (lesbian, gay, bisexual, transgender, queer/questioning, and additional identities) populations continue to face significant barriers to equitable sexual and reproductive health care. In Greece, legislative reforms have expanded rights for same-sex couples and gender-diverse individuals; however, these changes are not consistently reflected in everyday healthcare practices. Midwives and obstetric staff play a critical role in shaping access to inclusive and affirming sexual and reproductive health services.

This narrative review examines the structural, cultural, and professional factors influencing sexual and reproductive healthcare experiences among LGBTQ+ communities in Greece and, where relevant, in broader European and international contexts. A narrative literature review was conducted using PubMed and Google Scholar, focusing on peer-reviewed studies and relevant policy or legal sources published in English and Greek between January 2014 and March 2025. Search terms addressed LGBTQ+ populations, sexual and reproductive healthcare, midwifery, obstetric practice, healthcare access, provider education, discrimination, stigma, and inclusive care. Findings were organised thematically. Five interrelated barriers were identified: discrimination, stigma, and heteronormativity within healthcare encounters; insufficient education and training of midwives and obstetric staff regarding LGBTQ+ health needs; economic constraints and insurance-related exclusions; gaps between legal protections and implementation in clinical settings; and geographic disparities limiting access to inclusive services. These factors frequently intersect and reinforce inequalities in care provision.

Improving sexual and reproductive health outcomes for LGBTQ+ populations requires coordinated action beyond legal reform, including LGBTQ+-inclusive curricula, continuing professional development, respectful collection of sexual orientation and gender identity data, institutional accountability mechanisms, and equitable access to fertility, gender-affirming, preventive, and perinatal care services.

## Introduction and background

The LGBTQ+ community includes individuals with diverse sexual orientations and gender identities [1]. The acronym refers to lesbian, gay, bisexual, transgender, and queer or questioning people, while the plus sign encompasses additional identities not explicitly represented in the acronym [2]. Despite legal and social progress in many settings, LGBTQ+ people continue to experience substantial inequities in health, particularly in sexual and reproductive health (SRH) access and quality of care [3-11]. These inequities are shaped not only by individual-level factors, but also by systemic features of healthcare delivery, including cis-heteronormative assumptions, gaps in professional education, and non-affirming clinical environments [5,6,12-17].

In maternity and reproductive care, midwives and obstetric staff increasingly care for same-sex couples pursuing parenthood, including through assisted reproduction [6]. While positive experiences are frequently reported, LGBTQ+ service users also describe homophobia expressed through inappropriate questioning, avoidant communication, and reduced engagement from healthcare professionals [6]. Stereotypes about LGBTQ+ family structures and the role of the co-parent remain common, and a lack of consistent legal and institutional recognition may intensify stress and vulnerability during perinatal care [6].

Transgender and gender-diverse individuals report multiple barriers to SRH services, including contraception, fertility care, cancer screening, and abortion-related care [3]. Evidence indicates that discriminatory provider behaviors, limited provider training, and administrative systems that fail to accommodate gender diversity contribute to delayed care seeking and reduced quality of care [3,8]. In addition, the collection, or absence, of sexual orientation and gender identity (SOGI) data in routine healthcare settings influences visibility, continuity of care, and the ability of systems to monitor and improve quality for LGBTQ+ populations [15,18-22].

Recent legislative and policy developments in Greece reflect progress in LGBTQ+ rights. On 15 February 2024, the Greek Parliament passed legislation legalising same-sex marriage and granting adoption rights to same-sex couples [23,24]. However, evidence from broader European and international literature suggests that legal reforms do not automatically translate into equitable everyday healthcare experiences, particularly in SRH contexts [3,5,10]. A survey by the European Union Agency for Fundamental Rights reported discrimination in healthcare among LGBTI people, with higher levels reported by transgender respondents [10]. Consistent with these findings, recent systematic reviews highlight financial barriers, insufficient provider knowledge, and pervasive discrimination as recurring determinants of inequitable SRH care [3,19].

In response, this narrative review examines the accessibility and inclusivity of SRH services for LGBTQ+ people, with particular focus on obstetric and midwifery care. It aims to describe systemic and interpersonal barriers, including discrimination, provider education, economic constraints, legal and policy context, and geographic inequities, and to identify strategies to promote inclusive, equitable, and affirming reproductive healthcare. Emerging challenges in the contemporary information environment, such as SRH-related misinformation, may further undermine trust in services and complicate access for marginalised populations, reinforcing the need for evidence-informed, rights-based approaches [20-22,25,26].

## Review

Methods

Literature Search

A narrative literature review was conducted by the authors using PubMed and Google Scholar. These databases were selected because they provide broad coverage of biomedical, nursing, midwifery, public health, and policy-related literature relevant to sexual and reproductive health (SRH) and LGBTQ+ populations. The review was not designed as a systematic review or meta-analysis, and no claim of exhaustive evidence retrieval is made.

Searches were conducted for publications issued between January 2014 and March 2025. The search strategy used grouped Boolean combinations rather than isolated broad terms. Population terms included “LGBTQ”, “LGBTQ+”, “LGBTIQ”, “sexual minority”, “gender minority”, “transgender”, “gender diverse”, “non-binary”, “same-sex couples”, and “LGBTQ parents”. Healthcare-context terms included “sexual and reproductive health”, “reproductive healthcare”, “midwifery”, “obstetric care”, “maternity care”, “perinatal care”, “contraception”, “fertility care”, “assisted reproduction”, “cancer screening”, “abortion care”, and “gender-affirming care”. Barrier and quality-related terms included “healthcare access”, “quality of care”, “inclusive care”, “affirming care”, “discrimination”, “stigma”, “heteronormativity”, “provider education”, “training”, “insurance”, “rural”, and “geographic disparities”. Terms were combined using AND/OR operators and adapted to each database. Where relevant, “Greece” and “Europe” were added to identify region-specific literature.

The search was limited to English- and Greek-language publications. Peer-reviewed empirical studies, systematic reviews, narrative reviews, clinical education articles, and relevant policy or legal sources were considered eligible when they directly informed the quality, accessibility, or inclusivity of SRH care for LGBTQ+ populations.

Study Selection

Titles and abstracts were initially screened for relevance, defined as explicit engagement with LGBTQ+ populations and with issues related to access, quality, inclusiveness, barriers, or experiences in obstetric, midwifery, perinatal, or broader reproductive healthcare. Full-text articles were reviewed when eligibility could not be determined from the abstract alone or when the study appeared to address relevant healthcare experiences but did not clearly specify the care setting. Reference lists of included publications were manually screened to identify additional relevant sources. In keeping with narrative review methodology, inclusion was not restricted to a single study design. Eligible sources included quantitative observational studies, qualitative studies, mixed-methods research, systematic or scoping reviews, clinical guidelines, policy reports, and relevant grey literature, provided that they contributed directly to the review aims. This flexible approach allowed integration of diverse evidence on systemic and interpersonal barriers, service accessibility, provider education, discrimination, and inclusive care practices across heterogeneous healthcare contexts [11-17].

Inclusion and Exclusion Criteria

Studies were eligible for inclusion if they addressed sexual and/or reproductive healthcare for LGBTQ+ populations and were published in peer-reviewed journals, or if they provided directly relevant policy, legal, or methodological context. Eligible publications included qualitative, quantitative, and mixed-methods studies, as well as systematic and narrative reviews examining clinical practice, patient experiences, healthcare provider education, and structural or policy-related barriers to care.

Studies were excluded if they were opinion-based without empirical, policy, legal, or methodological relevance; not peer reviewed; unavailable in full text; or did not explicitly address sexual or gender minority populations. Grey literature and conference abstracts without full-text availability were excluded unless they provided essential policy or legal context directly relevant to Greece or LGBTQ+ healthcare rights.

Data Extraction and Synthesis

Data were extracted by the authors and included author/year, setting or context, source or study type, population or focus, healthcare setting, and main findings related to access, quality of care, provider knowledge and training, discrimination, and affirming practice. Because of the heterogeneity of included sources, findings were integrated narratively rather than statistically.

No formal risk-of-bias or quality appraisal tool was applied. This decision reflects the narrative design and the diversity of included evidence, which comprised qualitative studies, reviews, policy documents, legal commentary, and clinical education literature. No meta-analysis, meta-regression, pooled effect estimate, P value, or confidence interval was calculated. Instead, findings were synthesised thematically to identify recurring patterns, gaps, and intersections within the literature.

Results

Discrimination and Stigma in Sexual and Reproductive Health Services

Sexual and reproductive health constitutes a fundamental component of overall well-being; however, LGBTQ+ individuals continue to face disproportionate challenges when accessing SRH services. These challenges include economic and legal constraints, cultural stigma, and attitudes characterised by prejudice, discrimination, and insufficient education among healthcare professionals [5,10,20,25]. As a result, many LGBTQ+ individuals delay or avoid routine healthcare, conceal their sexual orientation or gender identity, and report reduced trust in healthcare systems [3,5,7,10,22,23,25].

Recent literature consistently identifies discrimination as a central barrier to the quality of SRH services for LGBTQ+ populations. Service users describe experiences of non-inclusive language, heteronormative assumptions, intrusive or irrelevant questioning, and lack of respect for gender identity and sexual orientation during healthcare encounters [5,6,12,20]. Such experiences are strongly associated with delayed care-seeking, reduced utilisation of preventive services, and poorer SRH outcomes [2,3].

Evidence suggests that discriminatory behaviours may range from subtle interpersonal interactions to overt denial of care. Transgender and gender-diverse individuals, in particular, report higher levels of mistreatment within healthcare settings compared with cisgender sexual minority groups [2,10]. A recent systematic review demonstrated that discrimination against transgender individuals negatively affects access to contraception, fertility services, cancer screening, and abortion-related care [2]. Similar findings have been reported in qualitative studies, where LGBTQ+ service users described healthcare encounters as emotionally unsafe or retraumatising, leading to long-term avoidance of SRH services [12,24,26-28].

Discrimination is further reinforced by structural features of healthcare systems that privilege cisgender and heterosexual norms. Cis-heteronormative clinical protocols, documentation, and communication practices contribute to the invisibility of LGBTQ+ identities and perpetuate exclusion within SRH services [5,7,15,25,26]. In obstetric and midwifery contexts, assumptions regarding heterosexual partnerships and binary gender roles remain common, often marginalising LGBTQ+ patients and their families [6,12,20].

Collectively, these findings highlight that discrimination and stigma are not isolated incidents but systemic determinants of inequitable SRH care. Their persistence undermines both the quality and accessibility of services for LGBTQ+ populations and represents a significant barrier to equitable sexual and reproductive health care.

Training and Knowledge of Healthcare Professionals

Inadequate education and training of healthcare professionals are consistently identified as key determinants of reduced quality in sexual and reproductive health (SRH) care for LGBTQ+ populations. Even in settings where providers express positive attitudes toward sexual and gender minorities, limited knowledge and lack of clinical competence often result in inappropriate care, ineffective communication, and unmet health needs [6,19,25].

Several studies report that healthcare professionals frequently associate LGBTQ+ health primarily with sexually transmitted infections, overlooking broader SRH needs such as contraception, fertility care, cancer screening, and perinatal support [8,25]. This narrow framing reinforces stereotypes and contributes to fragmented care delivery. LGBTQ+ service users have repeatedly reported being required to educate healthcare providers about their identities and specific health needs, a burden that undermines trust and shifts responsibility away from the healthcare system [8,20,25].

Within obstetric and midwifery care, gaps in education appear particularly pronounced. Although midwifery principles emphasise dignity, respect, and holistic, person-centred care, many LGBTQ+ individuals report negative experiences during maternity and reproductive care encounters [6,12,20]. Obstetric services often operate within heteronormative frameworks that assume heterosexual relationships and binary gender identities, resulting in inappropriate language, incorrect assumptions about partners, and exclusionary documentation practices [5,6,12,26]. These practices persist even when patients explicitly disclose their sexual orientation or gender identity [12,20].

Evidence from undergraduate and postgraduate education indicates that LGBTQ+ health topics are either minimally covered or entirely absent from midwifery and nursing curricula [8,19]. Studies suggest that the average amount of formal teaching time dedicated to sexual orientation and gender identity in healthcare education is limited, often insufficient to prepare providers for real-world clinical encounters [8,19]. As a consequence, healthcare professionals may feel uncertain, embarrassed, or fearful of causing offence, leading to avoidance of relevant discussions and reliance on stereotypes rather than evidence-based practice [21,25].

Training deficiencies are particularly evident in the provision of SRH care to transgender and gender-diverse individuals. Research highlights substantial knowledge gaps related to hormone therapy, fertility preservation, pregnancy in transgender men, and the impact of medical transition on reproductive outcomes [3,14]. Midwives frequently report limited clinical exposure to transgender patients and insufficient guidance on providing affirming perinatal care, further compounding uncertainty and discomfort in practice [3,14].

Importantly, evidence suggests that targeted education and LGBTQ+-inclusive training can significantly improve provider knowledge, attitudes, and confidence, leading to better communication and higher quality SRH care [21]. These findings underscore the central role of education in addressing disparities and highlight the need for systematic integration of LGBTQ+ health content into healthcare curricula and continuing professional development.

Access to Sexual and Reproductive Health Services

LGBTQ+ individuals experience significantly greater barriers to accessing sexual and reproductive health (SRH) services compared with the general population. These barriers are multidimensional and include financial constraints, lack of insurance coverage, institutional discrimination, and fear of stigma within healthcare settings [7,10,20,25]. Such factors often interact, compounding inequities and limiting timely access to essential SRH services.

Economic barriers represent a major determinant of reduced access to care. Sexual and gender minority populations are more likely to experience economic insecurity, unemployment, and lack of employer-sponsored health insurance, which directly affect their ability to access SRH services, including fertility treatments and gender-affirming care [7,17]. Transgender individuals, in particular, report delaying or forgoing healthcare due to cost-related concerns, especially when services are not covered by public or private insurance schemes [2,17,24].

In addition to financial constraints, institutional and administrative barriers further restrict access. Healthcare systems frequently lack clear pathways for LGBTQ+-inclusive SRH services, and administrative processes may fail to accommodate diverse gender identities and family structures [5,15,26]. Such barriers discourage service use and contribute to fragmented care experiences. Evidence also suggests that misinformation and inadequate communication regarding SRH services may exacerbate access difficulties, particularly in digital health environments where reliable, inclusive information is not consistently available [4,21,22].

Legal and policy contexts play a critical role in shaping access to SRH services. Although legislative reforms have expanded rights for LGBTQ+ individuals in several countries, including Greece, gaps in policy implementation and enforcement persist [26]. The absence of comprehensive legal protections for same-sex families and transgender individuals may intensify vulnerability and discourage engagement with SRH services, particularly during sensitive periods such as pregnancy or fertility treatment [13,26].

Geographic disparities further compound access barriers. LGBTQ+ individuals living in rural or remote areas often face limited availability of specialised or affirming SRH services and may be required to travel long distances to receive appropriate care [17,23]. These challenges disproportionately affect transgender and gender-diverse populations, for whom knowledgeable providers and inclusive clinical environments are less likely to be available outside metropolitan centres [17, 14].

Collectively, these findings demonstrate that access to SRH services for LGBTQ+ populations is shaped by intersecting economic, institutional, legal, and geographic factors. Addressing these barriers requires coordinated policy efforts, improved insurance coverage, the dissemination of accurate information, and the development of inclusive service models that ensure equitable access across diverse settings.

Affirming and Inclusive Care

Affirming and inclusive care has been identified as a critical indicator of quality in sexual and reproductive health (SRH) services for LGBTQ+ populations. Affirming care refers to healthcare practices that actively recognise, respect, and support individuals’ sexual orientation, gender identity, and family structures, rather than merely adopting a neutral or “one-size-fits-all” approach [15,18].

Evidence consistently demonstrates that LGBTQ+ individuals report higher satisfaction, increased trust, and greater utilisation of SRH services when care is delivered within affirming clinical environments [15,27]. Key elements of affirming care include the use of correct names and pronouns, inclusive language, respect for self-identified gender and relationships, and the creation of visibly safe healthcare spaces [18,27]. In contrast, failure to acknowledge LGBTQ+ identities-or treating them as irrelevant-may leave service users feeling invisible, uncomfortable, or inadequately supported, particularly in sensitive SRH contexts [15,20].

In obstetric and midwifery settings, affirming care plays a pivotal role in shaping perinatal experiences. LGBTQ+ individuals have reported instances in which midwives and obstetric staff either ignored their sexual orientation or gender identity or engaged in behaviours perceived as homophobic, biphobic, or transphobic, including inappropriate comments and dismissive attitudes [6,12,20]. Such encounters may undermine emotional safety and compromise the quality of care provided during pregnancy, childbirth, and the postnatal period [6,9,12].

Affirming practices are also closely linked to institutional structures and documentation. Clinical forms, electronic health records, and patient intake processes that rely on binary gender categories or heteronormative assumptions may inadvertently exclude LGBTQ+ individuals and limit opportunities for personalised care [5,15,27]. Research suggests that healthcare settings that systematically integrate sexual orientation and gender identity (SOGI) data collection in respectful and voluntary ways are better positioned to deliver coordinated, high-quality SRH services and to monitor equity in care provision [15,27].

Importantly, affirming care is not solely dependent on individual provider attitudes but requires organisational commitment, clear institutional policies, and ongoing professional training [18,21]. Studies indicate that when affirming practices are embedded at both interpersonal and structural levels, LGBTQ+ service users experience improved continuity of care and reduced avoidance of SRH services [27]. These findings underscore the importance of affirming and inclusive approaches as central components of equitable, patient-centred SRH care.

Structural and Legal Barriers

Structural and legal factors remain central determinants of inequalities in sexual and reproductive health (SRH) care for LGBTQ+ populations. Despite progress in legal recognition and anti-discrimination frameworks in several countries, the absence of explicitly inclusive healthcare policies and inconsistent enforcement of existing legislation continue to shape fragmented and exclusionary care pathways [5,10,26].

Legal reforms, such as the recent legalisation of same-sex marriage and the extension of adoption rights to same-sex couples in Greece, represent important milestones in advancing LGBTQ+ rights [26]. However, evidence suggests that such legislative progress does not automatically translate into equitable healthcare experiences. Limited institutional guidance, lack of provider awareness of legal changes, and absence of operational protocols within healthcare settings may restrict the practical impact of these reforms on SRH service delivery [13,26].

At the system level, the lack of routine collection of SOGI data contributes to the invisibility of LGBTQ+ populations within healthcare systems and constrains efforts to monitor quality of care and address inequities [15,27]. In addition, insurance policies and reimbursement frameworks may exclude or limit coverage for key SRH services, including fertility treatment, assisted reproduction, and gender-affirming care, thereby reinforcing disparities in access and outcomes [7,17].

Structural barriers are often compounded by deficiencies in professional education and organisational preparedness. Healthcare providers may be insufficiently trained to interpret and apply legal protections in clinical practice, particularly in contexts involving same-sex families or transgender individuals [21]. Consequently, LGBTQ+ service users may encounter uncertainty, administrative delays, or inconsistent treatment when accessing SRH services, even in jurisdictions with formal legal protections [5,10].

Overall, these findings indicate that structural and legal barriers operate alongside interpersonal discrimination to sustain inequities in SRH care. Addressing these challenges requires alignment between legal frameworks, institutional policies, professional training, and healthcare delivery systems to ensure that legal recognition is reflected in everyday clinical practice.

Geographic Inequalities

Geographic location represents an additional and significant barrier to equitable access to sexual and reproductive health (SRH) services for LGBTQ+ populations. Evidence indicates that specialised and LGBTQ+-affirming healthcare services are disproportionately concentrated in urban and metropolitan areas, while rural and remote regions often lack providers with relevant training or experience [17,23].

LGBTQ+ individuals residing outside major urban centres frequently face limited choice of healthcare providers, longer travel distances, and increased financial and time-related burdens when seeking SRH care [17]. These challenges are particularly pronounced for transgender and gender-diverse individuals, for whom access to knowledgeable and affirming providers is essential but often unavailable in non-metropolitan settings [17].

Geographic barriers may also exacerbate existing social and economic vulnerabilities. Travel requirements, confidentiality concerns in small communities, and fear of stigma may discourage individuals from seeking care altogether, leading to delayed diagnosis, reduced preventive care utilisation, and poorer SRH outcomes [14]. For some LGBTQ+ individuals, especially those with limited financial resources, geographic isolation represents a decisive factor in avoiding SRH services.

Collectively, these findings highlight that geographic inequities intersect with legal, economic, and institutional barriers to shape access to SRH care for LGBTQ+ populations. Addressing geographic disparities requires targeted policy interventions, expansion of inclusive services beyond urban centres, and investment in training healthcare professionals in rural and underserved areas.

The included evidence and contextual sources are summarised in Table 1. Collectively, these sources support six recurring themes: discrimination and stigma, provider education and training, service access, affirming and inclusive care, structural and legal barriers, and geographic inequalities.

**Table 1:** Overview of included literature and contribution to the thematic synthesis The evidence synthesised in Table 2 is drawn from references [1-28]. The key findings are summarized in the Appendices. SOGI: Sexual orientation– and gender identity; LGBTIQ+: lesbian, gay, bisexual, transgender, intersex, queer/questioning, and additional ; identities; SRH: sexual and reproductive health

Study	Setting/context	Source or study type	Population/focus	Main contribution to this review
Akre et al., 2025 [1]	Primary care; international/US context	Empirical study	Sexual and gender minority patients in primary care	Describes affirming activities in routine care and supports the importance of inclusive clinical practices and SOGI-responsive services.
Allen et al., 2025 [2]	International	Systematic review	Transgender and gender-diverse people	Identifies barriers to contraception, fertility care, cancer screening, and abortion-related care.
Almvik et al., 2023 [3]	Norway	Qualitative study	Midwives providing antenatal care for pregnant trans men	Highlights uncertainty, limited experience, and training needs in trans-inclusive antenatal care.
Alonso et al., 2024 [4]	Rural and LGBTIQ+ populations	Review/perspective on barriers	Rural and LGBTIQ+ service users	Supports the discussion of rural access barriers and misinformation affecting SRH access.
Bartels et al., 2025 [5]	Healthcare utilisation contexts	Systematic review	LGBTQ+ populations	Shows how cis-heteronormativity shapes healthcare utilisation and exclusion.
Brown et al., 2023 [6]	UK and Ireland	Education-focused study	Pre-registration midwifery programmes	Demonstrates limited LGBTQ+ health content in midwifery curricula.
Dahlhamer et al., 2016 [7]	United States	Population-based survey analysis	Adults identifying as sexual minorities	Documents healthcare access barriers among sexual minority adults.
Echezona-Johnson, 2017 [8]	Obstetrical nursing education	Educational evaluation	Nursing students/obstetrical nursing education	Shows gaps in LGBT knowledge within basic obstetrical nursing education.
Ellis et al., 2014 [9]	Maternity care context	Qualitative study	Male and gender-variant gestational parents	Describes conception, pregnancy, and birth experiences among gender-diverse parents.
European Union Agency for Fundamental Rights, 2020 [10]	European Union	Policy/survey report	LGBTI people in Europe	Provides comparative evidence on discrimination and inequalities across healthcare settings.
Green et al., 2006 [11]	Narrative review methodology	Methodological article	Narrative literature reviews	Supports use of narrative review methodology for heterogeneous evidence.
Hammond, 2014 [12]	Maternity care	Qualitative/exploratory study	Same-sex couples	Reports same-sex couples’ experiences of maternity care and heteronormative assumptions.
IDEA, 2024 [13]	Greece	Policy/legal tracker	Greek democratic and legal context	Provides contextual information on recent legal developments in Greece.
Johansson et al., 2020 [14]	Midwifery/childbirth care	Qualitative study	Midwives supporting trans men during childbirth	Identifies practical and relational challenges in trans-inclusive childbirth care.
Kerppola et al., 2019 [15]	Nordic maternity and child healthcare	Qualitative/health services study	LGBTQ parents	Supports the need for inclusive maternity and child healthcare settings.
Leong, 2025 [16]	Contraceptive care	Review/clinical article	Sexual and gender minorities	Supports discussion of contraception as an SRH domain for sexual and gender minority populations.
MacDougall et al., 2024 [17]	Urban and rural areas	Empirical study	Transgender and gender-diverse adults	Documents geographic inequalities in access to knowledgeable and affirming providers.
Margolies and Brown, 2019 [18]	Nursing/clinical practice	Clinical education article	Healthcare providers treating LGBTQ patients	Supports cultural competence and respectful communication in clinical encounters.
McCann and Brown, 2018 [19]	Healthcare education	Systematic review	Undergraduate healthcare education and professional training	Shows insufficient inclusion of LGBT+ health issues in health professional training.
McCann et al., 2021 [20]	International midwifery care	Systematic review	LGBTQ+ people using midwifery care	Identifies negative and affirming experiences in midwifery services.
Mezzalira et al., 2025 [21]	Primary, SRH, and mental healthcare	Guideline/review article	Healthcare providers treating LGBTQ+ patients	Provides clinical guidance for affirming and culturally competent care.
Purnat et al., 2025 [22]	Digital information environment	Infodemiology study/review	LGBTQ+ healthcare access and misinformation	Supports discussion of SRH misinformation and trust in services.
Renner et al., 2021 [23]	Rural regions	Empirical study	Transgender, non-binary, and gender-diverse people	Identifies rural barriers, travel burden, and confidentiality concerns.
Saadat et al., 2024 [24]	International transgender health	Review	Transgender people accessing SRH services	Summarises barriers and facilitators to SRH service access for transgender people.
Stewart and O’Reilly, 2017 [25]	Nursing and midwifery	Integrative review	Nurses and midwives	Shows attitudes, knowledge, and beliefs influencing care for LGBTQ populations.
Tryfonidou, 2024 [26]	Greece	Legal commentary	Same-sex marriage legislation	Provides context on Greek legislation opening marriage to same-sex couples.
World Health Organization, 2023 [27]	Global policy context	Policy brief	LGBTQ+ populations	Supports rights-based and law-informed approaches to sexual health.
Parker et al., 2025 [28]	Aotearoa New Zealand	Qualitative study	LGBTQIA+ people seeking abortion care	Provides evidence on abortion-care experiences and the need for identity-affirming services.

Discussion

The findings of this narrative review demonstrate that multiple, interrelated barriers continue to undermine the quality and accessibility of sexual and reproductive health (SRH) services for LGBTQ+ populations. These barriers operate at interpersonal, institutional, and structural levels and persist despite recent legal and policy advances in several contexts, including Greece. Discrimination and stigma remain prominent determinants of inequitable care, shaping both healthcare experiences and long-term patterns of service utilisation.

Consistent with previous international evidence, the results highlight that discriminatory encounters, ranging from non-inclusive language and heteronormative assumptions to denial of care, are strongly associated with delayed help-seeking and avoidance of SRH services among LGBTQ+ individuals [2,5,10,20]. Transgender and gender-diverse populations appear to be disproportionately affected, experiencing higher levels of mistreatment and structural exclusion within healthcare settings [2]. These findings reinforce the notion that discrimination in SRH care is not limited to isolated interpersonal interactions but reflects deeper systemic norms embedded in healthcare delivery.

A central theme emerging from this review is the persistent lack of adequate education and training among healthcare professionals, particularly within obstetric and midwifery care. Despite professional commitments to dignity and person-centred care, many providers report insufficient preparation to address the specific SRH needs of LGBTQ+ individuals [6,19,25]. As documented in the Results, educational gaps contribute to uncertainty, discomfort, and reliance on stereotypes, which may compromise communication and clinical decision-making. Importantly, evidence suggests that inclusive and targeted training can substantially improve provider competence, confidence, and quality of care, underscoring education as a critical lever for change [21].

Barriers to access further intersect with discrimination and educational deficiencies. Financial constraints, lack of insurance coverage, and administrative obstacles continue to limit access to contraception, fertility services, cancer screening, and gender-affirming care for LGBTQ+ populations [7,17]. These challenges are intensified by misinformation and inconsistent communication regarding SRH services, which may further erode trust in healthcare systems and discourage engagement, particularly among marginalised groups [4]. Such findings align with broader public health literature emphasising the role of accurate, inclusive information in promoting equitable SRH outcomes.

Affirming and inclusive care emerged as a key protective factor within this review. When healthcare environments actively respect sexual orientation, gender identity, and diverse family structures, LGBTQ+ individuals report higher satisfaction, greater trust, and increased use of SRH services [15,27]. These findings suggest that affirming care extends beyond individual provider attitudes to encompass institutional practices, documentation systems, and organisational culture. Routine and respectful collection of sexual orientation and gender identity data may further support continuity of care and enable health systems to monitor and address inequities more effectively [15,27].

Structural and legal contexts also play a critical role in shaping SRH care experiences. Although legislative reforms, such as the legalisation of same-sex marriage in Greece, represent important progress, their impact on healthcare practice remains uneven [26]. Limited institutional guidance and variable implementation of legal protections may result in continued uncertainty and exclusion for LGBTQ+ service users. These findings echo broader European evidence indicating that legal recognition alone is insufficient to eliminate disparities without parallel reforms in healthcare policy, education, and service delivery [5,10,13].

Geographic inequalities further compound these challenges. LGBTQ+ individuals living in rural or non-metropolitan areas face additional barriers, including limited availability of knowledgeable providers, greater travel burdens, and heightened concerns regarding confidentiality and stigma [17,23]. For transgender and gender-diverse individuals, geographic isolation may represent a decisive barrier to accessing appropriate SRH care, reinforcing existing health inequities.

Taken together, these findings suggest that improving SRH care quality for LGBTQ+ populations requires a coordinated, multi-level approach. Legal protections must be accompanied by clear institutional policies, comprehensive professional education, and the systematic integration of affirming practices within healthcare settings. Addressing financial and geographic barriers is equally essential to ensure that advances in rights translate into meaningful improvements in everyday healthcare experiences.

Clinical and Policy Implications

The findings support several practical implications for SRH services. First, undergraduate and continuing education for midwives, obstetricians, nurses, and allied health professionals should include LGBTQ+-specific SRH content, with practical scenarios addressing contraception, fertility care, antenatal and postnatal care, cancer screening, abortion-related care, and gender-affirming care. Second, healthcare organisations should review intake forms, electronic health records, and communication practices to ensure respectful and voluntary collection of sexual orientation and gender identity data. Third, clinical services should adopt visible and operational policies supporting inclusive language, recognition of diverse family structures, and clear pathways for referral when specialised care is required.

At the policy level, legal recognition must be accompanied by implementation mechanisms within healthcare services. This includes staff training on relevant legal rights, equitable insurance and reimbursement policies, accountability procedures for discrimination, and expansion of inclusive SRH services outside major urban centres.

Future Directions

The findings of this review highlight several directions for future research and practice in the field of sexual and reproductive health (SRH) care for LGBTQ+ populations. Further empirical research is needed to examine both the short- and long-term consequences of barriers to SRH access, not only in relation to reproductive outcomes but also with regard to mental health, psychosocial well-being, and overall quality of life. A more nuanced understanding of these impacts would clarify how structural and interpersonal inequities translate into measurable health disparities.

Future studies should also prioritise subgroup-specific analyses within the LGBTQ+ community. Treating LGBTQ+ populations as a homogeneous group risks obscuring the distinct experiences and SRH needs of transgender men, non-binary individuals, bisexual women, and LGBTQ+ people with intersecting identities related to ethnicity, migration status, disability, or socioeconomic disadvantage. Disaggregated data are essential to inform targeted and equitable interventions.

In addition, there is a clear need for the development and evaluation of structured educational interventions for healthcare professionals, particularly in obstetrics and midwifery. Such interventions should move beyond awareness-raising to include practical skills, clinical scenarios, and sustained professional development aimed at delivering affirming, evidence-based SRH care. Collaborative research involving both healthcare providers and LGBTQ+ service users could enhance the relevance and effectiveness of these interventions by grounding them in lived experience.

Finally, future research should explore the effectiveness of policy and system-level interventions, including the integration of sexual orientation and gender identity data collection, expansion of inclusive services in non-urban areas, and strategies to counter SRH-related misinformation. Evaluating how these approaches influence access, quality, and patient experience will be essential for translating policy commitments into meaningful improvements in care.

Limitations of the Review and Research Gaps

Several limitations should be considered when interpreting the findings of this narrative review. First, the literature search was limited to PubMed and Google Scholar. Although these databases provided broad biomedical and public health coverage, relevant studies indexed only in Scopus, Web of Science, CINAHL, PsycINFO, or other specialised databases may have been missed.

Second, the review was limited to English- and Greek-language publications, which may have excluded relevant evidence from other linguistic and healthcare contexts. The focus on literature from January 2014 to March 2025 ensured contemporary relevance but may have limited inclusion of older studies documenting the historical evolution of LGBTQ+ SRH care.

Third, because the review was narrative rather than systematic, exact database-specific hit counts, duplicate-removal counts, and exclusion counts were not prospectively recorded. A formal Preferred Reporting Items for Systematic Reviews and Meta-Analyses (PRISMA) flow diagram could therefore not be reconstructed without risking inaccurate reporting. This limits full reproducibility and should be considered when interpreting the scope of the evidence.

Fourth, no formal risk-of-bias or quality appraisal tool was applied. The included sources were heterogeneous and included qualitative studies, reviews, legal and policy sources, and clinical education literature. The findings should therefore be interpreted as a thematic narrative synthesis rather than as a graded assessment of evidence certainty.

An important research gap identified in this review is the frequent tendency to conceptualise the LGBTQ+ community as a single, uniform group. This approach risks overlooking the diverse and intersecting needs of subpopulations, particularly transgender, non-binary, and gender-diverse individuals, and LGBTQ+ people with intersecting vulnerabilities related to ethnicity, migration status, disability, socioeconomic disadvantage, or geographic isolation.

## Conclusions

This narrative review underscores the critical role of healthcare professionals, particularly midwives and obstetric staff, in delivering inclusive and high-quality sexual and reproductive healthcare to LGBTQ+ populations. Despite meaningful progress in legal recognition and policy frameworks, substantial gaps remain in the accessibility, acceptability, and inclusivity of SRH services. Discrimination, inadequate professional education, financial and geographic barriers, and inconsistent implementation of legal protections continue to compromise care for LGBTQ+ individuals.

Healthcare professionals have an ethical responsibility to provide informed, evidence-based, respectful, and equitable care regardless of sexual orientation or gender identity. Delivering human-centred and affirming SRH care requires individual professional commitment as well as structural support through education, institutional policies, accountable service design, and health system reform. In practical terms, this includes LGBTQ+-inclusive curricula, continuing professional development, inclusive documentation systems, voluntary and respectful SOGI data collection, equitable access to fertility and gender-affirming care, and expansion of inclusive services beyond metropolitan settings.

Legal reform is necessary but not sufficient. Its benefits will be realised only when rights are translated into everyday clinical practice through trained professionals, inclusive institutions, and accessible services. Aligning healthcare environments with principles of dignity, inclusivity, and affirmation is therefore essential for reducing SRH disparities and improving health and well-being among LGBTQ+ populations.

## Appendices

**Table 2:** Consolidated thematic findings on the quality of sexual and reproductive health (SRH) services for LGBTQ+ populations Consolidated thematic findings on the quality of sexual and reproductive health (SRH) services for LGBTQ+ populations

Category	Key Synthesised Findings	References (Vancouver)
Discrimination & Stigma	LGBTQ+ service users frequently report discriminatory language, heteronormative assumptions, lack of respect for sexual orientation and gender identity, and emotionally unsafe healthcare environments. Such experiences are associated with delayed care-seeking, avoidance of SRH services, reduced preventive care utilisation, and poorer SRH outcomes, particularly among transgender and gender-diverse individuals.	[2], [5], [10], [12], [20], [28]
Training of Healthcare Professionals	Insufficient LGBTQ+-specific education and limited curricular coverage in nursing, midwifery, and medical training contribute to clinical uncertainty, communication barriers, and inappropriate care. Targeted education and inclusive training programmes improve provider knowledge, attitudes, confidence, and patient trust, thereby enhancing SRH care quality.	[6], [8], [19], [21], [25]
Access to SRH Services	Sexual and gender minorities experience disproportionate barriers to accessing contraception, fertility services, cancer screening, and abortion care due to financial constraints, lack of insurance coverage, institutional discrimination, misinformation, and restrictive administrative processes. These barriers intersect and disproportionately affect transgender and socioeconomically disadvantaged populations.	[2], [7], [16], [17], [22]
Affirming & Inclusive Care	Affirming SRH care—characterised by correct use of names and pronouns, inclusive language, respect for diverse family structures, and safe clinical environments—is associated with higher patient satisfaction, increased service utilisation, improved continuity of care, and better SRH outcomes. Institutional commitment and inclusive documentation systems are critical for sustaining affirming practices.	[1], [15], [18], [27], [28]
Structural & Legal Barriers	The absence of explicitly inclusive healthcare policies, inconsistent implementation of legal protections, limited provider awareness of legal frameworks, and lack of systematic sexual orientation and gender identity (SOGI) data collection reinforce SRH inequalities. Legal reforms alone are insufficient without parallel institutional and educational changes.	[1], [5], [10], [26], [27]
Geographic Inequalities	LGBTQ+ individuals living in rural or remote areas face limited availability of knowledgeable and affirming SRH providers, increased travel burdens, confidentiality concerns, and heightened fear of stigma. Geographic isolation particularly restricts access to care for transgender and gender-diverse populations, exacerbating existing inequities.	[4], [17], [23]
